# Landfill sites selection using MCDM and comparing method of change detection for Babylon Governorate, Iraq

**DOI:** 10.1007/s11356-019-05064-7

**Published:** 2019-05-01

**Authors:** Ali Chabuk, Nadhir Al-Ansari, Hussain Musa Hussain, Jan Laue, Anwer Hazim, Sven Knutsson, Roland Pusch

**Affiliations:** 1 Department of Civil Environmental and Natural Resources Engineering, Lulea University of Technology, 971 87 Lulea, Sweden; 2 Department of Environment Engineering, college of Engineering, University of Babylon, Babylon, Iraq; 3 Remote Sensing Center, University of Kufa, Kufa, Iraq; 4 Koya University, Koya, 46017 Iraq

**Keywords:** MCDM, Change detection, RSW, AHP, Landfill siting

## Abstract

Landfill site’s selection represents a complicated process due to the large number of variables to be adopted. In this study, an arid area (Babylon Governorate as a case study) was selected. It is located in the middle region of Iraq. In this area, the landfills do not satisfy the required international criteria. Fifteen of the most significant criteria were selected for this purpose. For suitable weight for each criterion, the multi-criteria decision-making (MCDM) methods were applied. These methods are AHP and RSW. In the GIS software 10.5, the raster maps of the chosen criterion were arranged and analyzed. The method of change detection was implemented to determine the matching pixels and non-matching pixels. The final results showed that there are two candidate locations for landfills for each district in the governorate (ten sites). The areas of the selected sites were sufficient to contain the cumulative quantity of solid waste from 2020 until 2030.

## Introduction

Selecting an adequate site for landfill is necessary to protect human and environment. To determine the proper site for disposal of solid waste optimally, the decision-makers need wide expertise to evaluate the lands within the study area that conforms to the requirements of environmental and scientific factors and the regulations and determinants of local and central governments. In addition, the selection site for a landfill should meet the following factors like rapid economic growth, social, population growth rate, improvements in living standards, growing environmental awareness, and government and municipality funding (Siddiqui et al. 1996; Lin and Kao 1999; Javaheri et al. 2006).

Different effective techniques were used for disposition of the municipal solid waste in terms of solid waste management. Examples of these techniques are landfills, recycling, biological treatment, and thermal treatment (Kontos et al. 2003; Moeinaddini et al. 2010). The landfill is considered the most common technique that is adopted in various countries because this process is a relatively cheap and simple method to be used. In developed countries, after recycling large parts of their waste, the remaining materials are still to be dumped in the proper location (Yesilnacar and Cetin 2008; Kim and Owens 2010).

The general outlines to be considered for the best design for the chosen landfill locations are:

- Managing the waste disposal in a sound way for short and long-terms by reducing negative impacts on environmental factors (water, soil, and air) and the risk to human health.
- Inhibiting groundwater and surface water contamination by the leachate from landfill sites.
- Eliminating the effects of burning waste on human and surrounding environment.
- Controlling the gas emissions from landfill.
- Reducing the negative impacts on the environment and human population (Ireland EPA 2000; Scott et al. 2005).

In Babylon Governorate, the existing landfill sites do not fulfill the international criteria like that adopted in developed countries. In 2013, the solid waste generated in this governorate was 483,221 tonnes and the solid waste generation rate was 0.67 kg/capita/day. The budget spent on this process in that year was 15,894,716 USD (Chabuk et al. 2015). Groundwater depth in Babylon Governorate is shallow, which represent the main problem on human and environment when selecting the systematic sites for landfill. The water table depths in the whole area in the governorate vary from 0.423 to 15.97 m below the surface of the ground (Iraqi Ministry of Water Resources 2015). To solve the issue of selecting proper sites for landfill, the combination of the GIS software and multi-criteria decision-making (MCDM) methods can be used, which represent a quick way to achieve this purpose. The GIS software has an important role for the analysis of the input data and producing the required data for the landfill siting, since it has a high capability to deal with big different sizes of data (Kontos et al. 2003; Delgado et al. 2008; El Alfy et al. 2010; Şener et al. 2011). The GIS software and MCDM methods were applied to determine the best sites for landfills in each district in Babylon Governorate (Chabuk et al. 2016; Chabuk et al. 2017a, b, c, d, e).

Multi-criteria decision-making methods were used to derive the weights of criteria for the selected criteria. Then, these weights were applied on the maps of criteria in the GIS, after giving the suitable weights for the categories in each criterion map, to produce a suitable landfill site. Examples of such methods which were used in the current study are analytical hierarchy process (AHP) and ratio scale weighting (RSW).

Analytical hierarchy process (AHP) is a preferred method in multi-criteria decision-making methods. Thomas Saaty originally developed it in 1980. It is used to estimate the consistency weightings of criteria that resulted from constructing the matrix of pair-wise comparisons. In literatures, several studies used the analytical hierarchy process (AHP) method with GIS software in their studies to determine the weightings of criteria in the selecting sites for landfills (Siddiqui et al. 1996; Gemitzi et al. 2007; Ersoy and Bulut 2009; Eskandari et al. 2012; Kara and Doratli 2012; Alavi et al. 2013; Uyan 2014).

The ratio scale weighting (RSW) was used to obtain the weightings of criteria by allocating a proportion value for each criterion that is deserved through the decision-makers based on previous studies in this field and the opinion of experts.

In previous studies, several candidate landfill sites were identified in different areas using the ratio scale weighting (RSW) method and GIS (Halvadakis 1993; Sharifi and Retsios 2004; Sadek et al. 2006; Delgado et al. 2008; Nas et al. 2010). The change detection method was used in this study to compare the two output maps which were produced using the methods of AHP and RSW.

The purpose of this research is to obtain appropriate sites for landfill in Babylon Governorate, Iraq, using the ArcGIS 10.5 and the AHP and RSW methods (two methods of MCDM). In addition, the comparison method (change detection) was used to find the pixel percentage of matching and non-matching for the two raster maps of multi-criteria decision-making methods, and to verify the convenience of the chosen locations for the landfill on both output maps, AHP and RSW methods were used.

## Methodology

### Study area

**Fig. 1:**
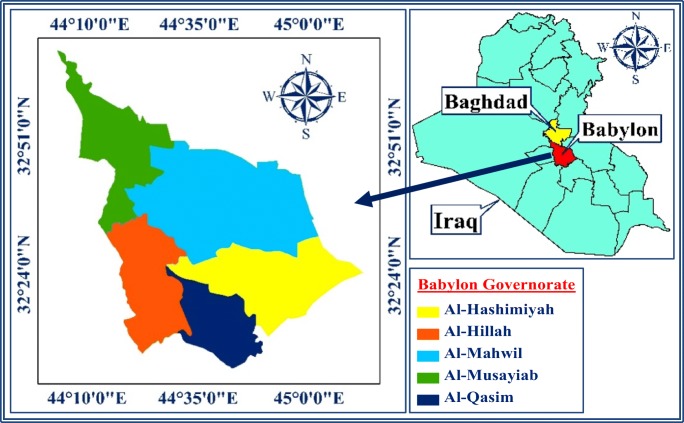
Babylon Governorate, Iraq

In the central part of Iraq, Babylon Governorate is located approximately 100 km to the south of Baghdad (capital of Iraqi) (Al Khalidy et al., 2010) (Fig. 1). It is situated between latitude 32° 5′ 41″ N and 33° 7′ 36″ N and longitude 44° 2′ 43″ E and 45° 12′ 11″ E (Fig. 1). Babylon Governorate has a rich history, and it contains several important archeological and religious sites, which includes one of the famous cities of the ancient world. The governorate has a population of about 2,200,000 up to the year 2017 and the inhabitants are distributed throughout its cities (Iraqi Ministry of Planning 2017). Babylon Governorate covers an area of 5337 km^2^. Administratively, Babylon Governorate is divided into five major cities (district) referred to as Qadhaa. These districts are Al-Hillah (capital of Babylon Governorate), Al-Hashimiyah, Al-Musayiab, Al-Mahawil, and Al-Qasim. These districts include 16 smaller cities, which are called Nahiah.

### Landfill siting model

**Fig. 2:**
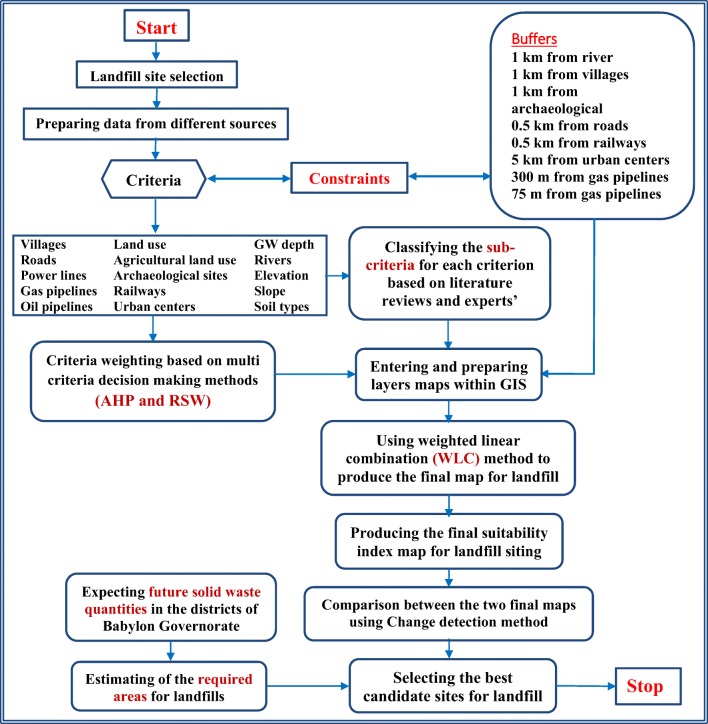
The model for landfill siting in Babylon Governorate, Iraq

The proposed model that will be led to obtaining the landfill sites in Babylon Governorate, Iraq, is summarized in Fig. 2**.**

### Site restrictions and buffer zones

In the site selection process for a landfill, important areas are excluded by giving them the rating of zero (e.g., agricultural land, orchards, archeological sites, industrial areas, universities, treatment plant, and airport). On the other hand, buffer zones should be created around or on both sides of specific geographic features for each criterion in the GIS software. Therefore, the site restrictions and buffer zones are necessary to protect the human health and to avoid any risk to the environment, as well as to fulfill the requirements of administrative regulations (Siddiqui et al. 1996; Ersoy and Bulut 2009). The buffer zones for urban centers, rivers, villages, roads, archeological sites, gas pipelines, oil pipelines, power lines and railways were created at distances of 5 km, 1 km, 1 km, 0.5 km, 1 km, 300 m, 75 m 30 m, and 0.5 km, respectively.

### Layers of the criteria maps

There are three sources used to prepare the required map layers in GIS software for the current study.

The first source was the vector data (shapefile feature classes) according to the internal reports of the Iraqi Ministry of Education (Iraqi Ministry of Education 2015). The first source was contributed to produce the separate shape file for urban areas, river, villages, elevation, slope, road, power lines, gas pipelines, oil pipeline, and railways.

In the second source, the relevant information in published maps was drawn as geometry features in separate polygons and storied their information and locations in shapefiles. The shapefile of “soil types” was obtained from the map of exploratory soil of Iraq (scale 1:1000, 000) (Buringh 1960). According to the World Digital Library (2013), the archeological map of Iraq (scale of 1:1500, 000) was used to identify the archeological and religious sites in Babylon Governorate. Then, the shape file of “archeological sites” was generated. The information on the published map (scale 1:1000,000) of “land capability map of Iraq” was converted to the shape file of “agricultural land use” according to the Iraqi Ministry of Water Resources (1990), and the categories of agricultural land use were verified by satellite images of Babylon Governorate. The published maps of industrial areas, treatment plants, and universities (scale 1:400, 000) (Iraqi Ministry of Municipalities and Public Works 2009) were used to define the locations of industrial areas, treatment plants, and universities within Babylon Governorate.

The third source includes the implementation of the spatial interpolation method “kriging” within spatial analysis tools in ArcGIS between defined readings. The map of groundwater depths was produced using this process where data from 185 wells distributed in the governorate (Iraqi Ministry of Water Resources 2015) were used.

In this study, all vector maps were converted to the raster maps to perform the analysis process for landfill siting in GIS.

### Determination of the sub-criteria weights

In this study, after analyzing the collected data for the 15 criteria, each criterion was classified into categories, and a value was given for each category as it deserves. This process was done based on the experts’ judgment, available data for the study area, and previous studies in this field. In this study, the geology criteria were ignored because there are no exposed rocks in this area, where alluvial deposits at depth of more than 50 m cover all the area of Babylon Governorate. Furthermore, Babylon Governorate is located outside the faults range (Jassim and Goff 2006). The 15 criterion and sub-criteria weights are presented briefly as follows:

#### Groundwater depth

**Fig. 3:**
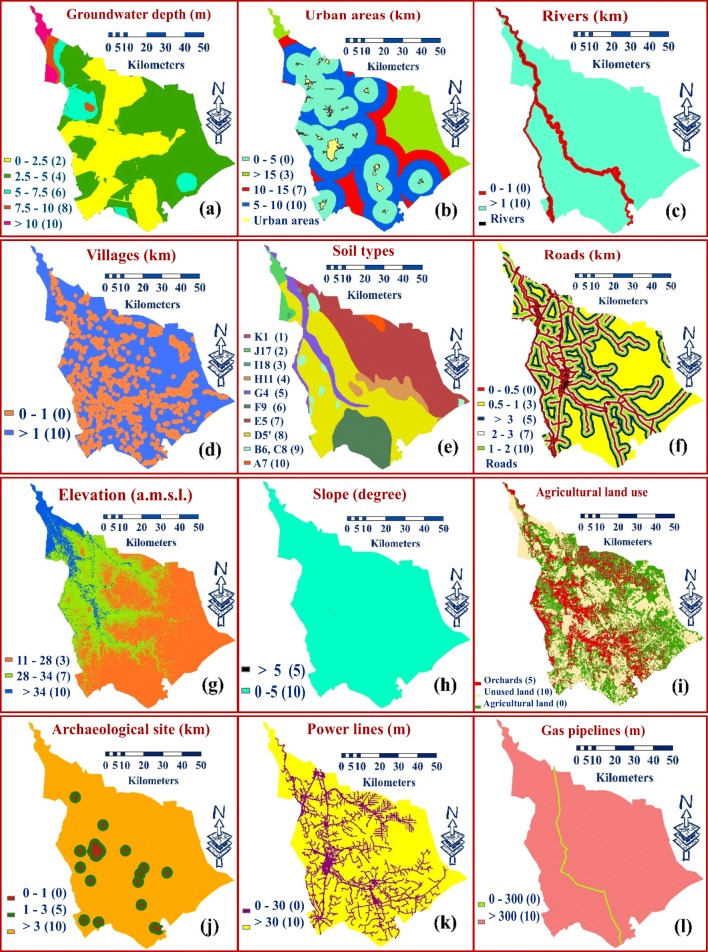
Classified maps of Babylon Governorate for **a** ground water depth, **b** urban center, **c** rivers, **d** villages, **e** soil types, **f** roads, **g** elevation, **h** slope, **i** agricultural land use, **j** archeological site, **k** power lines,**l** gas pipelines

The groundwater depth from the surface of the ground in most areas of Babylon Governorate is about0.42–15.97 m. The highest value of depth (deepest) was given the highest rating, while the lowest value of groundwater depth (shallowest) was given the lowest rating (Fig. 3a). In literature, many researchers suggested various depths from the landfill ground surface to the groundwater table (Table 1).

**Table 1:** The suggested depth of groundwater for the landfills according to previous studies

No.	Depth (m)	References
1	1.5	Alves et al. (2009)
2	6	Effat and Hegazy (2012)
3	10	Delgado et al. (2008)
4	15	Ouma et al. (2011)
5	30	Sadek et al. (2006)
6	2	Current study

#### Urban centers

In the current study, buffer zones of ≤ 5 km were assigned a rating of zero (Şener 2004; Effat and Hegazy 2012; Isalou et al. 2013). For buffer zones of 5–10 km and 10–15 km, the ratings of 10 and 7, respectively, were given. Buffer zones > 15 km were assigned a grading of 4 (Fig. 3b).

The classification of urban centers’ map was adopted to take into consideration the economic factors (transport and cost of land), as well as to protect the people and surrounding environment from negative impacts that results from the cumulative waste in landfills (e.g., diseases, insect, odors). Moreover, the potential to expand the urban areas in the future was also considered. In addition, there is always an increase of social opposition to establishing landfills (Zeiss and Lefsrud 1995; Tagaris et al. 2003; Effat and Hegazy 2012).

#### Rivers

Shatt Al-Hillah River is considered the main source for water in Babylon Governorate, and it passes through most of the cities of the governorate. For protecting surface water from contamination within the study area, a landfill site is not allowed to be established within the buffer distance ≤ 1 km on both sides of a river (Sharifi et al. 2009; Eskandari et al. 2012; Kara and Doratli 2012; Yildirim 2012). Any distance lower than 1 km, thus, was given a grading value of zero and any distance greater than 1 km was given a score value of 10 (Fig. 3c).

#### Villages

Due to the fact that there are high numbers of villages that are distributed throughout Babylon Governorate, buffer zones ≤ 1 km was allocated a score of zero (Charnpratheep et al. 1997; Şener 2004; Şener et al. 2006). Buffer zones ≥ 1 km were assigned a grading of 10 (Fig. 3d).

#### Soil types

There are eleventh types of soils in Babylon Governorate (Table 2 and Fig. 3e) (Buringh 1960). Alluvial deposits cover is about 50 m thick in Babylon Governorate (Jassim and Goff 2006).

**Table 2:** The soil types, symbol, and their rating weights in Babylon Governorate

No.	Soil type	Symbol	Rating
A	Periodically flooded soils	A7	10
B	Haur soils	B	9
C	Basin depression soils	C6	9
D	River basin soils, poorly drained phase	E5′	8
E	River basin soils, silted phase	D5	7
F	Silted haur and marsh soils	F9	6
G	River levee soils	G4	5
H	Active dune land	H11	4
I	Sand dune land	I18	3
J	Mixed gypsiferous desert land	J17	2
K	Gypsiferous gravel soils	K1	1

#### Roads

The layer of “roads” in the Babylon Governorate consists of the main roads and highway. In the current study, the distance of 0.0 to 0.5 km on both sides of the roads is considered buffer zone, and it was given a score of zero (Şener et al. 2006; Şener et al. 2011; Effat and Hegazy 2012). Buffer distances of 1–2 km, 0.5–1 km, 2–3 km, and > 3 km were given scores of 10, 7, 5, and 3, respectively (Fig. 3f).

Many factors should be taken into consideration for the roads’ criterion such as the economic factors regarding transport; the distances from roads to a landfill site should be appropriate to avoid the negative esthetic impacts. In addition, to avoid spending additional money as possible as through constructing new roads connecting the main roads with the selected locations for landfill should be considered (Zeiss and Lefsrud 1995; Lin and Kao 1999; Moeinaddini et al. 2010; Nas et al. 2010).

#### Elevation

In this study, the digital elevation model (DEM) was used (Iraqi Ministry of Education 2015). In Babylon Governorate, the elevation is ranging between 11 and 72 above mean sea level (a.m.s.l.). The criterion of elevation was selected for this study to decrease the possibility of leachate percolation through the landfill layers and to prevent the risk of seasonal flooding runoff (Demesouka et al. 2014). The elevation map of Babylon Governorate was separated into three classes. In the current study, the elevations of 34–72 m (a.m.s.l.) were assigned a score value of 10. Elevations between 28 and 34 m and 28 and 34 were and assigned values of 7 and 3, respectively (Fig. 3g).

#### Slope

The digital elevation model (DEM) of the study area was used to create the map of “slope.” Most of the land in the governorate has a slope of less than 5°, and it was given a score of 10 (Fig. 3h). The range of land slope for the current study is suitable for landfill siting through decreasing runoff of pollutants from the landfill to the surrounding areas (Lin and Kao 1999).

#### Agricultural land use

The “agricultural land use” map in Babylon Governorate includes three categories (agricultural, orchards, and unused). The category of unused land was given the highest possible score of 10. The “orchards” and agricultural land were given a value of 5 and zero, respectively. This is to protect agricultural land from destruction and contamination (Fig. 3i).

#### Archeological sites

In Babylon Governorate, there are many important archeological and religious sites. In this study, a value of zero was assigned for the buffer zones of ≤ 1 km from the archeological and religious sites in all directions (Gupta et al. 2003; Ersoy and Bulut 2009). A buffer zone more than 3 km around archeological and religious sites were assigned a value of 10, while buffer zones of 1–3 km were assigned 5 (Fig. 3j).

#### Power lines

For the power lines, on both sides, a buffer distance ≤ 30 m was assigned a rating of zero to avoid risks associated with high-voltage (Şener 2004; Yildirim 2012). Buffer zones higher than 30 m were given a score value of 10 (Fig. 3k).

#### Gas pipelines

For the map of “gas pipelines,” a grading value of zero was given for the buffer distance of less than 300 m from landfill sites to gas pipelines depending on the determinants of the Iraqi Ministry of Oil (2015). This distance was used to reduce the possible danger effect of fires that result from burning the waste on the pipelines of gas and oil. Buffer zone more than 300 m was given a score value of 10 (Fig. 3l).

#### Oil pipelines

**Fig. 4:**
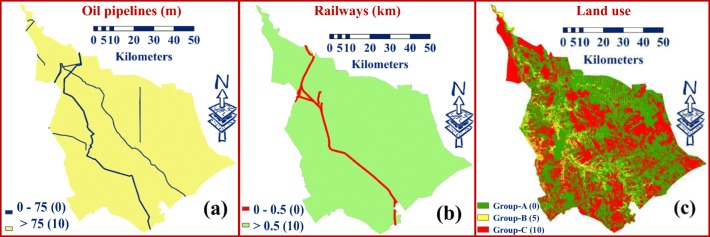
Classified maps of Babylon Governorate for **a** oil pipelines, **b** railways, **c** land use

The buffer zones more than 75 m on both sides for oil pipelines was given a score of 10 based on the determinants of the Iraqi Ministry of Oil (2015) on both sides of oil pipelines. A rating of zero was assigned for the buffer zones less than 75 m (Fig. 4a).

#### Railway

For the “railway” map, a rating of 10 was allocated to the buffer distances of ≥ 0.5 km on both sides of the railway. A value of zero was allocated for the buffer distance of ≤ 0.5 km (Wang et al. 2009; Nas et al. 2010; Demesouka et al. 2013) to avoid potential subsidence of land and visual intrusion (Baban and Flannagan 1998) (Fig. 4b).

#### Land use

To prepare the “land use” layer in Babylon Governorate, three groups were used. These groups are classified as A, B, and C. Group A includes archeological sites, rivers, universities, agricultural airport, treatment plant, industrial areas, agricultural land, urban centers, and villages. Group B contains plantation lands while group C comprises unoccupied lands. Figure 4c shows the ratings of 5 and 10 were allocated for groups B and C, respectively, while a value of 0 was allocated to all categories within group A.

### Multi-criteria decision-making methods

Two methods of MCDM were implemented to determine the criteria weights in dissimilar procedure. These methods are analytical hierarchy process (AHP) and ratio scale weighting (RSW). These methods can be summarized as follows:

#### Analytical hierarchy process method

Saaty (1980) developed the analytic hierarchy process (AHP) method. It is based on theoretical foundation. This method was used to derive the important weightings for the chosen criteria in the governorate, using a comparison’s matrix. The numerical scale of 9 points was used, where each point is used to express the relative importance between the two factors (Chabuk et al. 2017a).

**Fig. 5:**
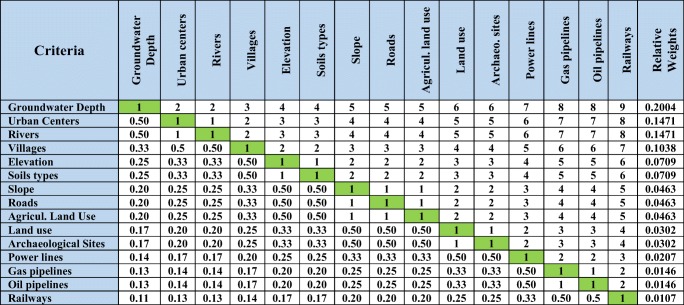
Pair-wise comparisons’ matrix for determining relative criteria weights for landfill siting

In the AHP method, the matrix of pair-wise comparisons was created to derive the relative weights of criteria (Fig. 5). In the AHP method, the eigenvector (Egi) was calculated for each criterion. Then, the eigenvalue for each criterion was normalized to produce the relative weights or the priority vectors (Pri) through dividing each eigenvalue by their sum (Chabuk et al. 2017a).

To check the consistency between the resultant weights for criteria, the value of the consistency ratio (CR) was computed through using the following formula: (CR = CI / RI) (Saaty 1980). In the current study, the value of the consistency index for the 15 criteria were 0.43, while the value of random index was 1.59 (see Table 3).

**Table 3:** Random inconsistency indices for different values of (*n* = 1, 2,..., 15) (Saaty 1980)

*n*	1	2	3	4	5	6	7	8	9	10	11	12	13	14	15
RI	0	0	0.58	0.9	1.12	1.24	1.32	1.41	1.45	1.49	1.51	1.48	1.56	1.57	1.59

The consistency is acceptable when the value of the consistency ratio is less than 0.1. Therefore, the CR value was 0.027 and smaller than 0.1.

#### Ratio scale weighting method

The second method which was applied in this study was the ratio scale weighting (RSW) method. In this method, the weights of criteria are given directly by decision-makers based on previous studies in this field. In this method, the decision-makers are giving a convenient proportional value for each criterion. A value of 100 is assigned to the most important criteria to be the base value for the other criteria. Each criterion is given the proportionally value smaller than 100 according to its importance relative to other criteria. The criteria are arranged proportionally from the most to the lowest significant (Şener 2004). Table 4 shows the weights of criteria for landfill siting using the RSW method.

**Table 4:** The criterion weightings defined for the RSW method and normalized weights (Chabuk et al. 2017d)

No.	Criteria	Ratio scale value	Standard weights (SW_*i*_)	Relative weights (RW_*i*_)
1	Groundwater depth	100	20	0.2012
2	Urban centers	74	14.8	0.1489
3	Rivers	73	14.6	0.1469
4	Villages	52	10.4	0.1046
5	Elevation	35	7	0.0704
6	Soils types	35	7	0.0704
7	Slope	23	4.6	0.0463
8	Roads	23	4.6	0.0463
9	Agricultural land use	23	4.6	0.0463
10	Land use	15	3	0.0302
11	Archeological sites	15	3	0.0302
12	Power lines	10	2	0.0201
13	Gas pipelines	7	1.4	0.0141
14	Oil pipelines	7	1.4	0.0141
15	Railways	5	1	0.0100
	Sum		99.4	1

To calculate the standard weights (SWi) in the ratio scale weighting (RSW) method, the proportional weight value for each criterion was divided by the value of proportional weight for the lowest importance criterion. The standard weights represent the new weights for each criterion.

The standard weights of criteria were converted to relative weights through divided each criterion standard weight by their summation, using Eq. (1) as follows (Chabuk et al. 2017d).

1 $$ {\mathrm{RW}}_i=\frac{{\mathrm{SW}}_i}{\sum_{j=1}^n\ {\mathrm{SW}}_j}\ j=1,2,n $$

where RW_*i*_ is the relative weight for each criterion; SW_*i*_ is the standard weights of each criterion of area *i* under criterion *j*; *n* is the criteria number.

## Results and discussion

### Final landfill siting maps

In order to implement the analysis process for producing the final map of landfills in GIS, all vector maps were converted to the raster maps. After producing the weightings for the 15 raster maps of criteria from AHP and RSW methods (Table 5), and the weights of categories for criteria, the special analysis tool “Map Algebra” in GIS software was applied to create the final raster maps of the suitability index for landfills, using Eq. (2) as follows.

**Table 5:** The relative weights of criteria calculated from the AHP and RSW methods

No.	Criteria	Relative weights	
		AHP	RSW
1	Groundwater depth	0.2004	0.2012
2	Urban centers	0.1471	0.1489
3	Rivers	0.1471	0.1469
4	Villages	0.1038	0.1046
5	Elevation	0.0709	0.0704
6	Soils types	0.0709	0.0704
7	Slope	0.0463	0.0463
8	Roads	0.0463	0.0463
9	Agricultural land use	0.0463	0.0463
10	Land use	0.0302	0.0302
11	Archeological sites	0.0302	0.0302
12	Power lines	0.0207	0.0201
13	Gas pipelines	0.0146	0.0141
14	Oil pipelines	0.0146	0.0141
15	Railways	0.0107	0.0100
	Sum	1	1

2 $$ {M}_i=\sum \limits_{k=1}^n{\mathrm{CW}}_k\times {\mathrm{SC}}_{ik} $$

where *M*_*i*_ is the index of suitability for area *i*; *n* is the number of criteria; CW_*k*_ is the relative weighting of each criterion; SC_*ik*_ is the rating value of area *i* under criterion *k*.

**Fig. 6:**
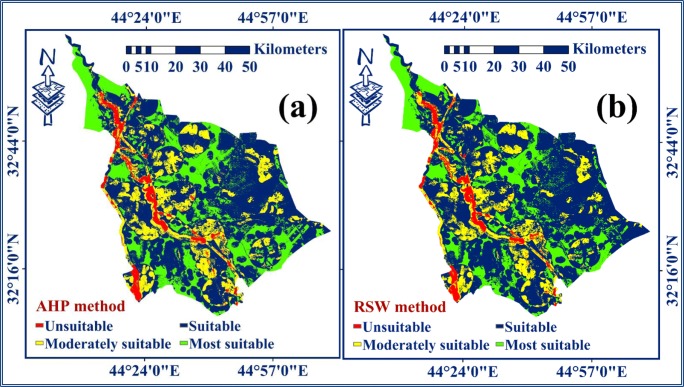
The maps of landfill siting in Babylon governorate using methods of **a** AHP and **b** RSW

Table 6 shows the summary of the number of pixels and the areas with their proportion for the maps’ categories for landfill siting, using AHP and RSW methods. Figure 6 represents the final raster maps of the suitability index of the selection sites for landfill using AHP and RSW.

**Table 6:** The pixels and the areas with their proportion for the categories in each map for landfill siting, using AHP and RSW

Category	AHP method			RSW method		
	No. pixels	Area (km^2^)	Proportion %	No. pixels	Area (km^2^)	Proportion %
USA	368,963	230.51	4.34	333,854	208.58	3.92
MDSA	1,397,117	873.50	16.42	1,237,999	773.59	14.54
SA	4,834,989	3022.67	56.82	5,251,135	3283.18	61.73
MSA	1,910,033	1192.62	22.42	1,688,114	1053.95	19.81

### Change detection method

According to Jin et al. (2013), the U.S. National Land Cover Database (NLCD) introduced the method of change detection. According to Chabuk et al. (2017d), the change detection method was applied for comparing the pixels of categories for the final raster maps in Babylon Governorate. In this study, each raster map was classified into four categories. The purpose of this method is to calculate the matching pixels for all categories and the non-matching pixels for every two similar categories for all categories.

**Fig. 7:**
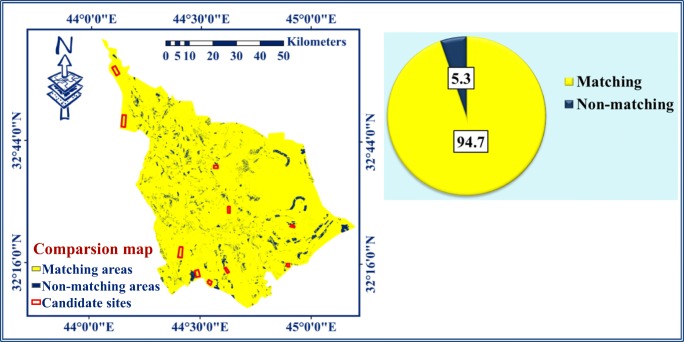
The comparison map for the AHP and RSW methods using change detection method

In this context, four categories exist. These are (i) most suitable areas (MSA), (ii) suitable areas (SA), (iii) moderately suitable areas (MDSA), and (iv) unsuitable areas (USA) (Chabuk et al. 2017d). In the GIS, the spatial analysis tool “Map Algebra” was applied using the formula “(AHP raster map) Diff (RSW raster map)” for comparison between the two maps using the change detection method. The process of comparison was applied to verify the appropriateness of the selected sites for landfill on the two resulted maps. The map of comparison resulted from combining the two final maps of AHP and RSW methods in Babylon Governorate (Fig. 7).

Table 7 shows the results obtained from the comparison map using the change detection method. It was noticed that there are two main categories, which are matching and non-matching areas for each two similar categories. In the comparison map (Fig. 7), the matching pixels’ ratio was 94.7% (in yellow), while the non-matching pixels’ ratio was 5.3% (blue). All selected sites for landfills are located within the category of matching areas on the comparison map.

**Table 7:** The data of comparison map that generated from combining of the methods of AHP and RSW

Value	Count	Categories (AHP)	Categories (SRS)	Pixel ratios	Classification
1	8,059,847	All similar categories	All similar categories	94.70	Matching
2	35,109	(USA) 4	(USA) 4	0.41	Non-matching
4	194,227	(MDSA) 3	(MDSA) 3	2.28	Non-matching
5	221,919	(MSA) 1	(MSA) 1	2.61	Non-matching

*USA* unsuitable areas, *MDSA* moderately suitable areas, *MSA* most suitable areas

### Selecting candidate sites for landfill

To calculate the quantity of waste produced for the year 2030 in Babylon Governorate and its districts, Eq. (3) was used for this purpose according to Chabuk et al. (2015).

3 $$ {\mathrm{SWQ}}_{(rt)\left(\mathrm{for}\ \mathrm{specific}\ \mathrm{year}\right)}=\left(\left({P}_{0(2013)}{\left(1+0.0299\right)}^n\right)\times \left({\mathrm{GRW}}_{(2013)}{\left(1+0.01\right)}^n\right)\times \left(365/1000\right)\right) $$

This equation was constructed based on three factors. These are:

(i) The annual increment rate of (1%) for waste generation rate (WGR) starting from the year 2013.
(ii) Solid waste generation rate for Babylon Governorate in 2013 (SWGR).
(iii) The expected population for the year (2030) was built based on the existed population in 2013 and using the annual growth rate of 2.99% (Iraqi Ministry of Planning 2013).

The solid waste cumulative quantity during the years from 2020 to 2030 in Babylon Governorate and its districts was estimated using Eq. (3) (Table 8). The solid waste cumulative quantity that generated by 2030 can be computed, using Eq. (4) as follows:

4 $$ {\mathrm{SWCQ}}_{(r)}={\mathrm{SWQ}}_{(rt)}+{\mathrm{SWCQ}}_{\left( rt-1\right)} $$

where SWCQ_(*r*)_ is the solid waste cumulative quantity for the particular year (tonne); SWQ_(*rt*)_ is the solid waste quantity for the particular year (tonne); SWCQ_(*rt−*1)_ is the solid waste cumulative quantity for the previous year before the particular year (tonne).

**Table 8:** The summary of computing the solid waste quantity in 2030, and the solid waste cumulative quantities for the years 2020–2030 (Chabuk et al. 2015)

District	Population, *P*_*o*(2013)_	Population, *P*_*t*(2030)_	Solid waste quantity SWQ (2013) (T)	SWGR (kg/capita day) (2013)	Solid waste quantity SWQ (2030) (T)	Solid waste cumulative quantity SWCQ (2020–2030) (T)
Al-Hillah	807,777	1,332,930	238,244	0.82	472,474	4,300,864
Al-Qasim	184,605	304,621	38,913	0.57	76,374	695,219
Al-Mahawil	336,148	554,685	49,377	0.4	96,389	877,419
Al-Hashimiyah	270,020	445,566	51,491	0.52	100,155	911,695
Al-Musayiab	374,684	618,274	105,196	0.77	205,792	1,873,295
Babylon Governorate	1,973,234	3,556,966	483,221	0.67	1,030,174	8,752,506

The volume of waste in the year 2030 and the cumulative waste volume from 2020 to 2030 in the governorate and its districts are shown in Table 9. These values were calculated based on the following information:

**Table 9:** The volume of waste in 2030 and its cumulative volume for the years 2020–2030 in Babylon Governorate and its districts

District	Waste volume in 2030 (m^3^)	Cumulative waste volume 2020–2030 (m^3^)
Al-Hillah	674,963	6,144,091
Al-Qasim	109,106	993,170
Al-Mahawil	137,699	1,253,456
Al-Hashimiyah	143,079	1,302,421
Al-Musayiab	293,989	2,676,136
Babylon Governorate	1,471,677	12,503,580

The data are given in Table 8.

Values of waste volume in 2030 are the result of dividing the solid waste quantity in 2030 and solid waste cumulative quantity for the years 2020–2030 by the density of waste (700 kg m^−3^) (Oweis and Khera 1998; Worrell and Vesilind 2002; UNEP-IETC 2006).

The required area of candidate sites for landfills was calculated through dividing the expected cumulative volume of solid waste generated from 2020 to 2030 in each district by the 2 m height of solid waste that will be placed on the top surface of the candidate sites. Then, the result of the required area in each district was multiplied by 10% to provide a factor of safety when selecting the candidate sites (Chabuk et al. 2015). The initial reasons of selecting the height of solid waste in these sites as 2 m are as follows:

- The groundwater depth in the study areas is shallow.
- To reduce the cost of constructing a perimeter berm around the sites.
- To reduce soil subsidence or settlement under the load of cumulative waste that will be placed over the surface at the selected sites.

In each district, two candidate sites were selected for landfill among many sites that were located within the category of the “most suitable.” The required areas and the selected sites’ areas for landfills in each district are tabulated in Table 10.

**Table 10:** The required areas, selected sites’ areas, and its locations for landfills in the Babylon Governorate districts of (Chabuk et al. 2017b)

District	Required area (km^2^)	Area of candidate sites		Location
		Site	Area (km^2^)	
Al-Hillah	3.4	Hi-1	6.768	Latitude 32° 15′ 46″ N
				Longitude 44° 28′ 55″ E
		Hi-2	8.204	Latitude 32° 13′ 43″ N
				Longitude 44° 29′ 15″ E
Al-Qasim	0.55	Q-1	2.766	Latitude 32° 11′ 43″ N
				Longitude 44° 32′ 26″ E
		Q-2	2.055	Latitude 32° 14′ 38″ N
				Longitude 44° 37′ 10″ E
Al-Hashimiyah	0.72	Hs-1	1.288	Latitude 32° 15′ 54″ N
				Longitude 44° 53′ 38″ E
		Hs-2	1.374	Latitude 32° 24′ 51″ N
				Longitude 44° 54′ 41″ E
Al-Mahawil	0.70	Ma-1	2.950	Latitude 32° 29′ 59″ N
				Longitude 44° 41′ 2″ E
		Ma-2	2.218	Latitude 32° 38′ 12″ N
				Longitude 44° 34′ 9″ E
Al-Musayiab	1.4	Mu-1	7.965	Latitude 32° 48′ 39″ N
				Longitude 44° 8′ 59″ E
		Mu-2	5.952	Latitude 33° 0′ 14″ N
				Longitude 44° 6′ 46″ E

**Fig. 8:**
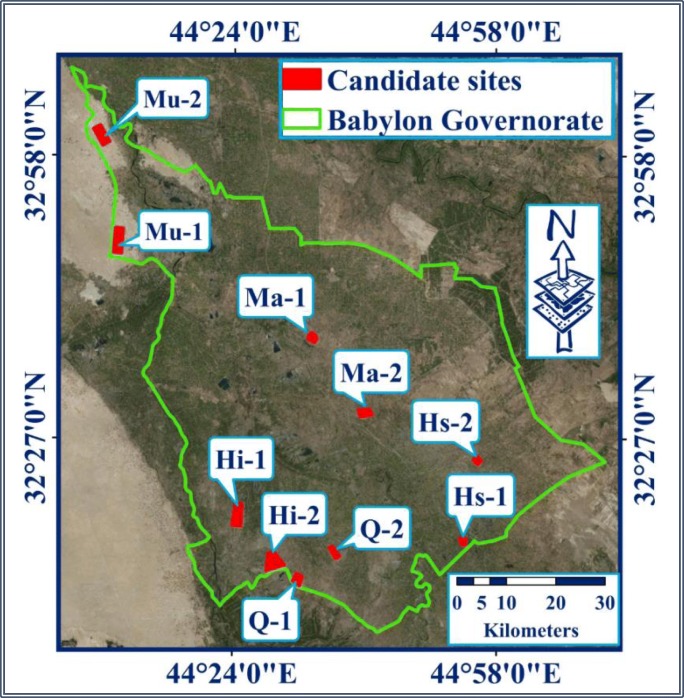
Candidate sites for landfill on the satellite images of the Babylon Governorate

For accuracy purposes, the selected sites were checked on the satellite images of the governorate to verify their suitability within the districts of Babylon Governorate (Fig. 8).

## Conclusions

The present waste disposal sites in Babylon Governorate do not conform to the environmental and scientific criteria, and it influences human health. The purpose of the current study is to select the most suitable landfill sites in the governorate using the GIS software (10.5) and methods of AHP and RSW. Thus, 15 maps of criteria were entered into the GIS to produce the final map for landfill siting. The 15 layers are groundwater depth, urban centers, rivers, villages, soil types, elevation, agriculture lands use, roads, slope, land use, archeological sites, gas pipelines, oil pipelines, power lines, and railways.

Two MCDM methods were implemented in different styles to find the relative weights for each criterion. The analytic hierarchy process (AHP) was the first method, where a matrix of pair-wise comparisons between each criterion to derive the weight to each criterion was used. The second method was the ratio scale weighting (RSW). This method is based on the experts’ opinion and previous studies in this field by giving proportion values for each criterion according to its importance among other criteria. Then, the special analysis tool in GIS “Map Algebra” was used to generate the final map to select the candidate sites for landfill for each method.

The two final maps that resulted from the two methods of MCDM (AHP and RSW) were combined in the GIS. Then, the change detection method was used to find the matching and non-matching areas on the final raster maps of AHP and RSW methods. The comparison process of the change detection method was applied to check the appropriateness of the landfill sites that were selected in Babylon Governorate on the two maps that were produced from the AHP and RSW methods.

Finally, ten candidate sites were obtained on the final maps for landfill in Babylon Governorate among several sites (two for each district). All the selected sites were located within the category of “most suitable” on the final maps of MCDM methods and within the matching areas in the comparison map. It was found that these sites are suitable to accommodate the cumulative solid waste for the years 2020–2030. This procedure will allow the planners and decision-makers to apply it in other areas in Iraq that have similar conditions (especially in the arid areas) when selecting a new landfill site.
